# Co-designing physical activity intervention in Danish kindergartens: A design-based research approach

**DOI:** 10.1371/journal.pone.0361522

**Published:** 2026-11-05

**Authors:** Mathilde Sederberg, Charlotte Skau Pawlowski, Lise Sohl Jeppesen, Elisabeth Bandak, Anna Bugge

**Affiliations:** 1 Department of Midwifery, Physiotherapy, Occupational Therapy and Psychomotor Therapy, University College Copenhagen, Copenhagen, Denmark; 2 Centre of Better Childhood, University College Copenhagen, Copenhagen, Denmark; 3 Active Living Research Unit, Department of Sports Science and Clinical Biomechanics, University of Southern Denmark, Odense, Denmark; VIVE - The Danish Center for Social Science Research, DENMARK

## Abstract

Physical activity in early childhood supports health, psychosocial development, and motor competence, yet many Danish kindergarten children do not meet recommended activity levels. Early childhood settings are therefore key health promotion environments where routines, pedagogical practices, and staff competencies shape children’s access to daily physical activity. This study examines how a kindergarten-based intervention can be co-designed to strengthen physical activity by equipping pedagogical staff and supporting enabling environments through participatory, practice-embedded processes using a design-based research approach. Five Danish kindergartens participated in a one-year development phase guided by a complex intervention framework. The process included four iterative domains: context analysis, laboratory work, design experiments, and reflection. Data from observations, interviews, workshops, and prototype testing were analyzed thematically. The co-design process resulted in six elements: active group time with PA prompts; a play catalog with pedagogical cues; a key ring with pedagogical prompts; structured playground activities; designated PA Agents; and a competence development program. Six themes informed the design: Physical activity as an overlooked pedagogical resource; variation in children’s physical activity competences; the role of leadership in cultural change; pedagogical staff’s bodily engagement; pedagogical judgment as a driving force, and; competence development as an adoption strategy. The process produced a contextually grounded and professionally owned intervention. By strengthening local capacity, ownership, and equitable access to physical activity, the co-design approach supports health promotion embedded in every day kindergarten practice and informs future feasibility testing.

## Introduction

Physical activity (PA) in early childhood is essential for health and development [1,2]. WHO recommends that children aged 2–4 years engage in at least 180 minutes of daily PA, including 60 minutes of moderate-to-vigorous physical activity (MVPA) [3]. Yet many children in Denmark and other Western countries do not meet these guidelines. For example, 22% of Danish 5‑year‑olds fail to achieve the recommended daily MVPA [4].

Danish kindergartens represent a key arena for promoting PA, as 98% of Danish children attend and spend most of their day there [5]. In Denmark, kindergartens typically enroll children aged 3–6 years and are part of the publicly funded early childhood education and care system. Groups of children are cared for by pedagogical staff, which primarily consists of trained pedagogues (holding a professional bachelor’s degree in early childhood education) and assistants with more varied educational backgrounds. Daily practice is characterized by a strong emphasis on play, outdoor activities, and relatively flexible routines, where children spend substantial time both indoors and in outdoor playground environments.

International reviews show that early childhood settings can effectively support PA [6,7]. However, PA levels vary greatly across kindergartens, highlighting the importance of pedagogical practices and local PA culture [8]. Research also suggests that externally led interventions are often more effective than those implemented solely by local pedagogical staff, underscoring the need for capacity building approaches [7]. Staff report barriers such as time constraints, limited competencies, and insufficient leadership support [9–11], pointing to a need for flexible, practice‑oriented approaches that reflect the complexity of everyday life and foster shared ownership [12]. Despite the potential of outdoor play, children remain largely sedentary during kindergarten outdoor hours where only 14% of time is spent in MVPA [13]. This emphasizes the need to integrate active play as a natural part of daily routines.

Pedagogical staff possess deep contextual knowledge and close relationships with the children and are therefore central in bridging research and practice. To realize this potential, the development of PA promoting interventions in kindergarten settings must involve a broad range of stakeholders, including pedagogical staff, leaders, municipal administration, and other key collaborators [14–16]. Yet limited knowledge exists on how they can be actively involved in the design and implementation of PA interventions in kindergartens [17,18]. Participatory, practice‑embedded, and contextually grounded approaches may be essential for integrating PA into daily routines. Such approaches build local professional capacity and support health promoting environments for young children [19].

Taken together, these perspectives underscore the importance of involving pedagogical staff and leaders directly in the development of PA promoting interventions. To address this gap, the Danish Moving Kindergartens project will co-develop an intervention aimed at increasing children’s PA during kindergarten hours in collaboration with pedagogical staff and leaders. In subsequent phases of the project, the intervention and its evaluation procedures will be tested in a feasibility study, followed by a larger-scale trial to assess the intervention’s effects on children’s PA. The present article focuses exclusively on the intervention development phase of the project and examines how kindergarten structures and pedagogical practices can be developed to support children’s PA.

## Materials and Methods

### Study design

The project follows the Medical Research Council’s (MRC) guidance for developing and evaluating complex interventions [20], which emphasizes an iterative and systematic research approach. Accordingly, the project includes three stages:

1. Development – designing the intervention and its theoretical foundation.
2. Feasibility testing – examining whether the intervention and evaluation procedures are feasible and acceptable
3. Evaluation – if feasibility is confirmed, conducting a large-scale cluster Randomized Controlled Trial (RCT) to test effectiveness.

The development phase ran from January to December 2025, followed by a 10‑week feasibility study from March to May 2026. The feasibility study will focus on testing the intervention elements and the feasibility of the evaluation procedures, while the effects on children’s PA will be assessed in the subsequent randomized controlled trial.

Pedagogical staff and leaders were actively involved in generating, adapting, and testing intervention elements through workshops, design experiments, and reflection activities. Participation varied across phases and included collaborative development, consultation, and feedback reflecting a dynamic understanding of participation in which stakeholders assume different roles and degrees of influence throughout the process [21]. Although the research team retained final responsibility for design decisions, participants informed these decisions through their professional and contextual knowledge. Thus, in this study, co-design is understood as an iterative and collaborative development process in which researchers and practitioners jointly contribute to shaping intervention elements. In line with Vargas et al. (2022), co-design refers to the active involvement of stakeholders in designing solutions to a predefined problem, distinguishing it from broader participatory approaches such as co-creation [22].

### Participants and recruitment

Five kindergartens varying in enrolment, type, and geographical location participated in the development phase (Table 1). Participants were recruited using purposive sampling through professional networks, based on the first author’s professional role as an experienced lecturer in early childhood education. Recruitment of kindergartens and participants took place from 01/12/2024–01/04/2025. The authors had no prior personal or professional relationship with the interviewed participants before study commencement. Initial contact was established through pedagogical consultants acting as gatekeepers in selected municipalities. All participants received written information about the Moving Kindergartens project prior to participation. The information described the overall aim and purpose of the study, the first author’s role as a researcher and lecturer within early childhood education, and the third author’s role as a researcher. The researchers involved had professional experience in early childhood education, interviewing, and facilitating development processes. No personal goals beyond the stated research objectives were disclosed. Participants included 2–3 pedagogical staff and 1 kindergarten leader from each of the five participating kindergartens. The pedagogical staff represented diverse professional backgrounds, including trained pedagogues with formal education in early childhood education and assistants with more practice-based training, ranging from newly employed staff to practitioners with many years of experience. Kindergarten leaders held responsibility for the overall pedagogical and organizational management of the institutions.

**Table 1:** Characteristics of the five kindergartens included in the development phase.

Kindergarten	1	2	3	4	5
Child enrolment	138	40	102	80	67
Geographical location	Suburb	Forest	Urban	Urban	Urban
Type	Public	Private	Public	Public	Public

### Domains for development and data collection

To guide the development of a kindergarten-based PA intervention within the overarching MRC framework for complex interventions, this study applied a Design-based Research (DBR) methodology [23,24] during the intervention development phase. Co-design constituted the participatory process, while the DBR approach was operationalized through four domains: Context, Lab, Design Experiments, and Reflections. Across these domains, intervention elements were generated, adapted, and tested iteratively. While the research team retained final responsibility for design decisions, the process was informed by participants’ contextual knowledge and insights of early childhood education and PA. These inputs guided the facilitation of workshops and the iterative refinement of intervention elements. Taken together, this approach enabled the integration of key intervention features identified in the literature, including structured elements, practice-based training, coaching, and iterative refinement through testing and adaptation.

DBR enables iterative, collaborative development that integrates theoretical insights with practitioners’ experiential knowledge and is well suited to complex educational settings requiring locally responsive solutions. The approach also aligns with contemporary health promotion principles by emphasizing participation, contextual tailoring, and collaborative knowledge production, key to fostering equitable and sustainable change in educational settings [19]. Fig 1 provides an overview of the development process across the four domains, adapted from the DBR model proposed by Jørgensen et al. (2021), and illustrates the iterative movement between contextual exploration, co-design, testing, and reflection throughout intervention development.

**Fig 1:**
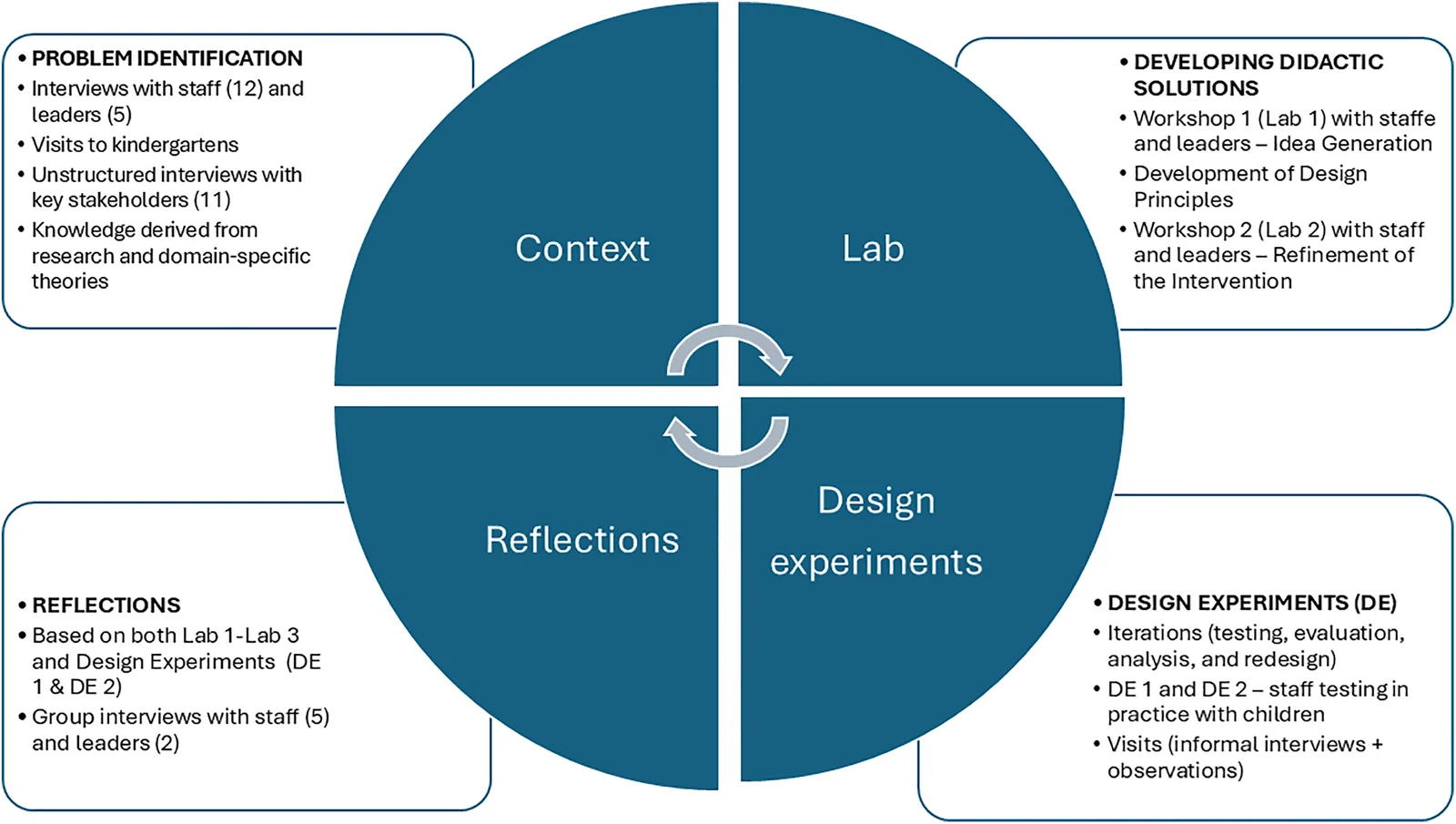
Overview of the design model inspired by Jørgensen et al. (2021), structured across four domains (Context, Lab, Experiment, and Reflection), and illustrating participant involvement across the different phases of the development process.

### Context domain

Within the Context domain, the aim was to capture the full complexity of the problem and to ensure that the intervention was firmly grounded in the practical realities of daily kindergarten practice. This involved combining knowledge from existing research (e.g.[6–10,18,25,26], with practical know-how from national kindergarten-based PA initiatives. Special attention was given to how various stakeholders, such as management and municipal authorities, influenced the pedagogical staff’s pedagogical practice in relation to PA.

Within this domain, the first author (MS) conducted initial semi‑structured face‑to‑face group interviews with nine pedagogues and three assistants from the five collaborating kindergartens, as well as five face-to-face semi‑structured individual interviews with the five leaders. The interviews were guided by a thematic interview guide structured around three research questions:

1. How did informants understand PA in the kindergarten context?
2. What was the current status of PA in their kindergarten (including pedagogical activities and structural conditions)?
3. What were the pedagogical staff’s wishes, needs, and ideas regarding PA in kindergarten?

The interview guide was developed based on both theoretical and practical knowledge, aiming to balance methodological rigor with openness to participants’ perspectives [27]. Although it was not formally pilot tested, the first interviews served as informal pilots and led to minor refinements of questions. Examples of questions from the interview guide are: How will you describe the concept of PA? How do you work with PA? What elements do you think are important for successful physical activities in kindergartens? Each interview lasted approximately 45–60 minutes and was carried out onsite in the kindergartens. All interviews were audio-recorded and subsequently transcribed. No one else besides the interviewer and the informants was present during the interviews. Audio recordings were transcribed using the transcription software GoodTape.

In addition, eleven unstructured interviews were conducted using purposive sampling with key stakeholders possessing specialized knowledge of early childhood education and/or PA (e.g., labor union representatives, municipal officials, movement consultants). Each interview was conducted by the first (MS) and third (LSJ) authors and lasted approximately 30 minutes. Six of the eleven interviews were conducted face-to-face at various locations, during which detailed notes were taken. The remaining five interviews were conducted via Microsoft Teams, audio-recorded with prior consent, and subsequently transcribed. The empirical data collected through these interviews was used to gain insight into opportunities, needs, and barriers related to PA practices in kindergartens.

### Lab domain

The Lab domain consisted of three collaborative workshops (Lab 1, Lab 2 and Lab 3), facilitated by the first (MS) and third (LSJ) authors, which served as the central arena for co-design. Based on insights from the context domain, four leaders and ten of the pedagogical staff participated in Lab 1 developing the core elements of the intervention. Three leaders and 8 pedagogical staff reconvened for the second workshop (Lab 2), where the intervention elements and strategies were refined and adjusted based on feedback and shared reflections [24,28]. Four participants withdrew due to organizational changes in the kindergartens. No one refused participation for reasons related to the study. Lab 3 was conducted with a leader, a pedagogical staff member, two key stakeholders, and three researchers. This session focused on identifying the core competencies considered essential for working with the intervention. All Labs were conducted at the researchers’ workplace in a creative workshop setting designed to support collaborative design processes.

In all Labs, empirical data were collected through audio-recordings of participants’ group discussions and presentations, in addition to wall posters summarizing key points from group processes.

### Design Experiments domain

The Design Experiments were carried out in two iterative cycles (DE 1 and DE 2). The aim was to examine how the intervention elements worked in practice and to establish a basis for adjustments. In DE 1, the initial intervention elements were tested over five months in the five participating kindergartens. DE 2 was conducted over two months to test the adjusted elements. These cycles allowed for continuous adaptation and learning, ensuring that the intervention remained grounded in the realities of pedagogical practice.

Participant observations, including unstructured field interviews, were conducted by the first and third (MS and LSJ) authors in DE 1 and DE 2 to examine actual PA practices and to gain insight into the pedagogical staff’s experiences with the intervention. By participating in the daily life of the setting, the researcher gained access to the nuanced, contextual dynamics that are essential for understanding how interventions are interpreted and adapted in practice [29,30]. During observations, detailed fieldnotes were taken using structured observation sheets with predefined focus points, such as how staff applied the intervention materials, their collaboration and staging of activities, use of space and attention to children’s participation.

### Reflections domain

The Reflections domain was integrated throughout the development process through ongoing dialogue and feedback. It culminated in the final development phase, where two face-to-face semi-structured group interviews were conducted by the first (MS) and third (LSJ) authors with two leaders and five pedagogues from the participating kindergartens. Guided by a semi-structured interview guide the aim was to provide insights into the participants’ experiences of engaging in the co-designed intervention developing process. Both interviews, each lasting approximately 30 minutes, were audio-recorded and transcribed. This domain supported both theoretical and practical development, helping to surface tacit knowledge and values embedded in everyday practice.

### Data analysis

All interview transcriptions and observation fieldnotes were analyzed using a thematic analysis approach [31]. The analysis was conducted manually by the first (MS) and third (LSJ) authors, while the remaining authors contributed through ongoing discussions and iterative refinement of codes and themes throughout the analysis process. Issues of data sufficiency were discussed within the research group to assess the adequacy and richness of the data material. In line with the iterative nature of the DBR approach, sufficiency was not defined as a fixed endpoint but was evaluated continuously throughout the development process. Data were considered sufficient when recurring patterns and themes across interviews, observations, and co-design activities provided a stable foundation for informing and refining intervention elements. In the later stages of the development phase, only limited new insights emerged, and the existing data were deemed adequate to guide subsequent design decisions and adaptations. Transcripts were not returned to participants, in line with the iterative DBR process where feedback was obtained through ongoing co-design workshops and reflections, where preliminary findings and intervention elements were discussed and refined. Initially, transcripts and fieldnotes were read multiple times to gain familiarity with the material. Codes were then generated inductively, capturing patterns related to participants’ experiences, practices, and contextual factors. These codes were iteratively grouped into broader themes that reflected central aspects of the co-design process and the context. The data collected in each domain (i.e., within the context domain, the Lab activities, the design experiments, and the reflection domain) were analyzed separately to inform subsequent decisions and adaptations during the process. The interpretation of findings and subsequent design decisions were informed through an interplay between practitioner perspectives and research-based knowledge, consistent with the collaborative and iterative nature of the DBR approach.

Given the study’s substantial use of semi‑structured interviews and researcher–participant interactions, we followed the COREQ 32‑item checklist to ensure transparent and rigorous reporting of the qualitative components. The completed checklist is provided in the Supporting Information (S1. COREQ Checklist).

### Ethics

All pedagogical staff members and leaders who participated in the co-design workshops and semi-structured interviews during the development phase provided informed written consent prior to participation. The information described the overall aim and purpose of the study, and the included researchers’ role in the project. Key stakeholders were also informed about the project before the unstructured interviews conducted with them. None of the key stakeholders, leaders or staff members received any form of financial compensation for their involvement. All data were anonymized prior to analysis and prior to inclusion in the research database accessed by the authors.

According to Danish regulations, informed consent from parents or children was not required, as no data were collected directly from children and the study focused exclusively on professional practices and organizational processes. The study was approved by the Research Ethics Committee at the University of Southern Denmark (REC), protocol number: 20250516.

## Results

This section presents the key findings illustrating how the intervention was developed through an iterative co-design process. The analysis is structured according to the four domains: Context, Lab, Design Experiments, and Reflections. Within each domain, data were examined using thematic analysis [31], focusing on what emerged as most significant in that domain for shaping the intervention. Findings are presented in relation to their data sources and how they informed the development of intervention elements. To provide an overview of how findings across domains informed the development of the intervention, Fig 2 presents the relationships between domains, analytical themes, design decisions, intervention elements, expected mechanisms, and intended outcomes across the iterative development process:

**Fig 2:**
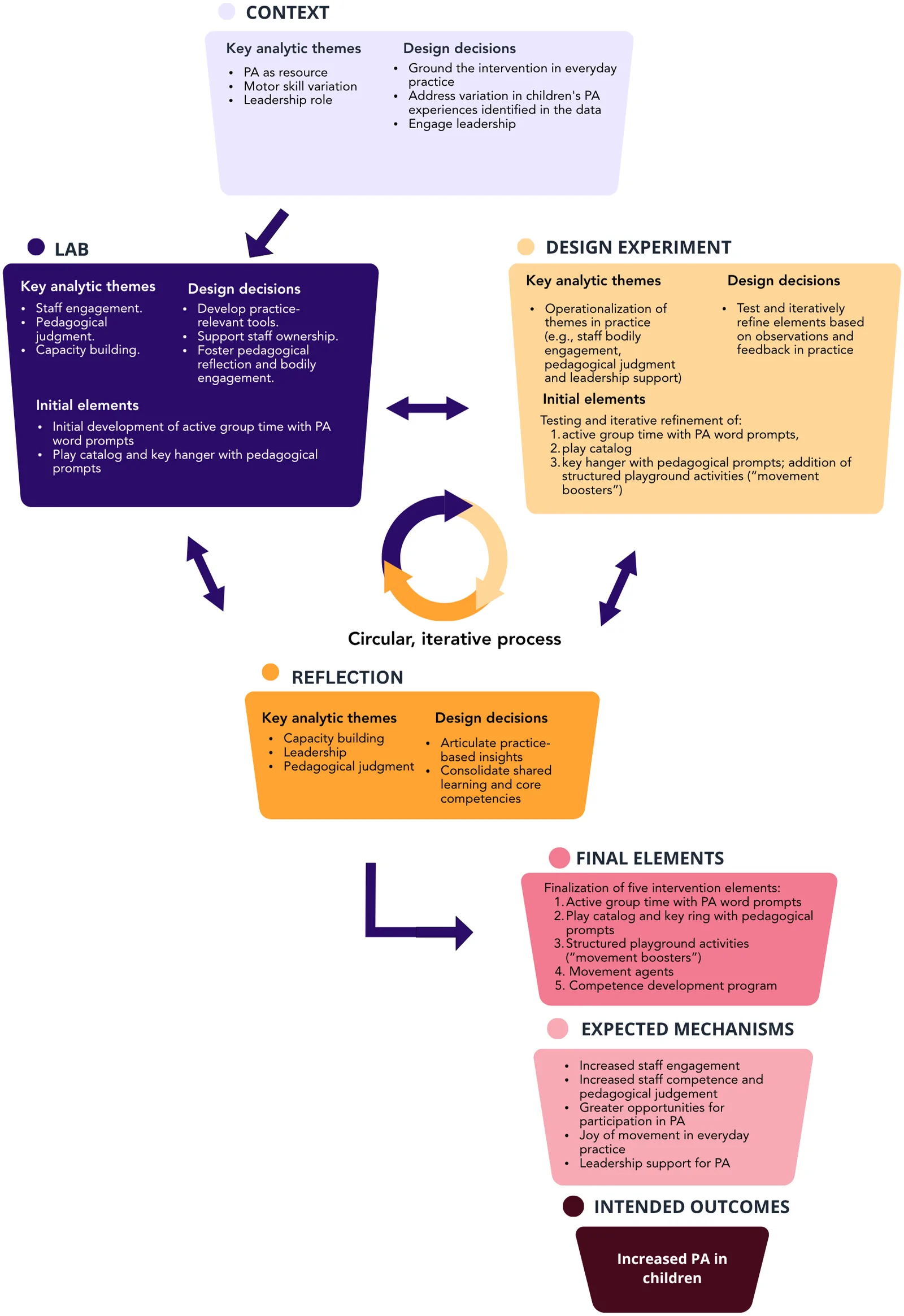
Relationships between domains, analytical themes, design decisions, intervention elements, expected mechanisms, and the intended outcome of increased children’s physical activity (PA) across the iterative Design-Based Research (DBR) process.

The figure shows how findings from each domain informed analytical themes, design decisions, and intervention elements throughout the development process. While the domains are presented separately in the following sections, the process was circular and recursive, with insights generated in later domains also contributing to the ongoing understanding of context and intervention development. The expected mechanisms represent pathways through which the intervention elements are anticipated to influence practice and children’s participation in PA, ultimately contributing to increased children’s PA in subsequent project phases. The following sections describe how findings from each domain contributed to the development of analytical themes, design decisions, and intervention elements.

### Context: Understanding practice and the problem

Analysis of the initial interviews revealed recurring patterns in pedagogical staff and leaders’ narratives that became decisive for the design. These patterns highlighted underlying tensions, variations, and conditions that shaped everyday practices and pointed to areas requiring attention for meaningful change. As illustrated in Fig 2, these contextual insights provided the foundation for subsequent design decisions and intervention development while also being continuously refined through later domains in the iterative design process.

The initial context exploration showed that PA is often deprioritized in the kindergarten’s daily routine and rarely included as a strategic pedagogical focus. PA was described by several informants as secondary compared to, for example, language learning activities: “Why do we keep going along with that political agenda that it should be language, when our children are calling for something else?” (Leader, Kindergarten 3). At the same time, PA was highlighted as an inclusive approach, particularly for children with linguistic or social challenges: “Much of that [PA] they can participate in without language (...). So, the more we can get these community games going (...), the more we can make them part of the community”. (Leader, Kindergarten 4).

The interviews and observations pointed to large local variations in children’s PA competencies. Several informants described children as motorically inexperienced:” They [the children] are motorically inexperienced. They have no idea if they can (...) I actually think many of them aren't as motorically poor as we think. They simply just haven't learned it yet” (Staff, Kindergarten 2). This statement underscores the need for structures that support all children’s participation in PA and provide adaptable opportunities for engagement across different levels of PA competence. This informed the later development of flexible and adaptable intervention elements. At the same time, it was emphasized that it is central to involve children’s ideas in the development of play focusing on PA competencies: “I think it’s really important that we are curious together, the children and me.” (Staff, Kindergarten 5).

Finally, both leaders and pedagogical staff emphasized that leadership is crucial for translating PA initiatives into everyday practice. As one leader explained: “Change requires close leadership. Staff must have a clear understanding of the expectations they are required to meet.” (Leader, Kindergarten 1). Likewise, a staff member emphasized: “It is important that we have understanding and backing [from the leader]. But it is also important that leaders see the bigger picture and support the initiative with adequate resources” (staff 5, Kindergarten 6). Recognizing the critical role of leadership, the project actively involved pedagogical leaders in the development phase. The need for key pedagogues in the staff group who can support colleagues and strengthen the quality of PA was also pointed out: “Selected key persons should simply create a customized action plan (...), also for those people who don't have this expertise.” (Staff, Kindergarten 5).

Together, these findings informed the initial analytical themes related to PA as a pedagogical resource, variation in children’s PA competencies, and leadership engagement, which subsequently guided intervention development (Fig 2).

### Lab 1: Idea Generation of the Intervention’s Core Elements

In workshop 1, participants formulated concrete ideas based on the challenges that emerged from the Context domain and led to following specific ideas:

- “Treasure chest” with PA words
- The kindergarten’s own play catalog
- Key ring with pedagogical prompts

These initial intervention ideas emerged directly from the analytical themes identified in the Context domain and represent the first operationalization of the iterative design process illustrated in Fig 2. The ideas functioned as initial design propositions that were subsequently developed, adapted, and refined through workshops and testing in practice.

The first design sketches of the intervention’s elements emerged from discussions about “body and PA ” and “staff’s practice and competencies.” Participants emphasized that pedagogical staff must actively demonstrate joy in the physical activities to engage children: “Well, that we ourselves demonstrate joy in what we’re doing.” (Staff, Kindergarten 1). This highlighted that bodily engagement and enthusiasm cannot be assumed: “It’s kind of assumed that [pedagogical engagement] is just there, but it isn’t just there.” (Staff, Kindergarten 3). These findings highlighted the importance of pedagogical staff’s bodily engagement and joy of PA in the intervention design. Accordingly, this informed the development of intervention elements aimed at supporting staff’s active participation and role modelling in everyday practice. A set of PA words was developed for the “treasure chest” intended as a tool to support active group time [a structured group activity] in kindergartens. The idea was to create a playful resource that could introduce daily PA prompts and inspire variation in activities. As a staff member explained as part of an idea presentation:

“We imagine a “treasure chest” that recurs. So every day there is something new in it (…) it could be today’s movement. Like falling, dancing, spinning, etc. So, if today was spinning, how can you spin around yourself? What can we find that can spin? Can fingers spin? Everything. There could also be rhythm and togetherness if it has something to do with dancing (…) You could also guess today’s word if there were lots of words. We are still brainstorming if we stand here.” (Staff 3, Kindergarten 2).

This concept became a central design idea for engaging children in PA through curiosity and play during group time. As shown in Fig 2, this element was linked to design decisions aiming to strengthen everyday opportunities for children’s participation in PA.

The play catalog was initially developed from pedagogical staff’s descriptions of favorite and frequently used games gathered during Lab 1. These games served as practice-based examples of meaningful and feasible PA activities in kindergarten settings.

The idea of the “key ring with pedagogical prompts” emerged through collaborative design activities. The concept aimed to create small, portable play guides with open-ended questions to involve children as co-developers, such as: “How do you think the game is going?”, “What works well?”, “What doesn’t work?”, and “What can we do to make the game better?” Pedagogical staff emphasized that these tools could foster shared learning with the children.

### Design Experiment 1 (DE 1): Intervention Elements as Structures for PA

DE 1 included testing of the three intervention elements developed in LAB 1: (1) “Treasure chest” with PA words during active group time (2) The kindergartens own play catalog, and (3) Key ring with pedagogical prompts. In line with the iterative process presented in Fig 2, DE 1 focused on testing and refining the intervention elements generated in Lab 1 through experimentation in practice.

The “treasure chest” with PA words tested during group time was highlighted as particularly engaging for both children and adults: *‘*It works well with the words and we put things in the chest ourselves. It creates a moment of excitement’ (Staff, Kindergarten 2). Another staff member mentioned: “They just want more. Several say ‘more’, and everyone has been involved the whole time.” (Staff, Kindergarten 2). This indicated that a simple play activity can function as a strong pedagogical driver for children’s engagement in PA when it is connected to a familiar structure such as group time. The play catalog was also tested, and the researchers and practitioners collaboratively reviewed, adapted, and expanded the activities drawing on knowledge related to children’s PA. The resulting catalog was organized around themes identified in Lab 1—such as rough-and-tumble play, PA competencies and sensory experiences, community-building, and PA storytelling. Designing the catalog around these widely applicable themes was crucial for feasibility, ensuring that the intervention could be adapted and implemented in other kindergartens without requiring major changes to their daily routines. Furthermore, the catalog was conceived as a flexible resource that could be expanded and tailored to each kindergarten’s own ideas and preferences, supporting both scalability and local ownership.

Testing of the play catalogue and the key ring revealed important findings for the intervention’s competence development element. Observations indicated that the play catalogue contained good games but was text-heavy and difficult to use without prior hands-on experience. As one staff member noted in an unstructured field interview: “We need to get better at using the play catalogue because we just pick the games we already know.” (Staff, Kindergarten 3). This highlighted the importance of trying all elements physically during competence development activities. Accordingly, the intervention was designed to include hands-on training, where pedagogical staff actively engaged with and practiced the use of the play catalogue and related activities. Similarly, the key ring was seen as a relevant methodological tool, but primarily for less experienced assistants rather than trained pedagogues:

“It’s probably mostly for less experienced assistants because as a pedagogue you have many of these questions in your backbone. It would be a good idea to have easy games first on the key ring and then the didactic questions.” (Staff, Kindergarten 1).

These findings emphasize that practical testing and adaptation are crucial for ensuring usability and relevance across staff roles. Accordingly, the key ring was developed to include both simple, accessible games and accompanying didactic prompts, allowing it to support less experienced staff while also facilitating reflection and child involvement in play development.

Pedagogical staff emphasized that these tools could foster shared learning with the children: “Then we can learn something together as a group.” (fieldnotes, staff, Kindergarten 4). This illustrates how pedagogical judgment shaped and adapted activities, ensuring ownership and flexibility within the intervention. Observations shows this in practice: “Gathering children for a quick tactical talk; “How do you think the game is going?” and “How can we catch them more easily?” which led to a boy suggesting spreading out as catchers, improving the play.” (fieldnotes, staff, Kindergarten 4). These prompts inspired the key ring concept, making child involvement systematic and accessible for all staff, reinforcing co-design at the center of the intervention. These findings illustrate how practical testing and staff feedback informed ongoing design decisions and refinement of the intervention elements across domains, as illustrated in Fig 2.

Another central finding in DE 1 was the importance of the pedagogical staff’s active participation and initiation of PA on the playground. Observations of play like “zombie -play” (a role-play) and dialogue with pedagogical staff showed how their use of facial expressions, energy, and intentional staging played a decisive role in supporting children’s participation: “It’s also very much about facial expressions and how you immerse yourself in a role as a zombie.” (Staff, Kindergarten 6). Here it became clear that the intervention is not only about activities but largely about the way pedagogical staff facilitate and participate in them. It became evident that playground time was a moment requiring attention: “There are many opportunities for movement on the playground. Some children are left on their own and struggle to join play communities?” (Staff, Kindergarten 5). In Kindergarten 7, they had tested different roles on the playground, including a ‘PA booster’, **a staff member assigned to initiate and facilitate a designated period of PA during playground time**. They also experimented with different scheduling formats to coordinate the initiative, which became a development point in Lab 2.

### Lab 2: Adjustment and Adaptation

Lab 2 functioned as an arena for joint evaluation and adjustment of the intervention elements that had been tested in DE 1. In particular, this workshop focused on developing a structure for increasing children’s PA during playground time. The playground roles as ‘PA boosters’ were discussed as having potential to strengthen children’s participation as well as pedagogical staff’s attention to children’s different participation opportunities in PA: “(...) I think we're precisely in a place that could come really far with something like this [organizing “PA boosters on the playground”].” (Leader, Kindergarten 1). Based on these discussions and workshop inputs, it was decided to test “PA boosters on the playground” in two of the Kindergartens in DE 2. This represents an example of how iterative testing and stakeholder feedback informed the development of new intervention elements across domains, as illustrated in Fig 2.

Further discussions focused on how to strengthen PA facilitation skills, leading to the addition of an intervention element termed “PA Agents”. PA Agents served as resource persons within each group and coordinated which staff members would act as PA boosters during playground time. Although a PA Agent could also take on the PA booster role, the two terms referred to distinct functions: PA Agents had a broader coordinating and collegial support role, whereas PA boosters were responsible for initiating and facilitating PA during designated periods on the playground. Conversations revealed that not all staff were equally enthusiastic or confident in initiating PA, and support from more experienced colleagues was considered crucial. The PA Agent role was therefore developed with a coaching dimension, focusing on supporting and inspiring colleagues to initiate and engage in movement activities in everyday practice. This included modelling engagement, facilitating activity initiation, and encouraging colleagues to experiment with and adapt activities to different groups of children. Through this role, PA Agents supported practice-based development of PA facilitation, with particular attention to children’s participation in PA activities. The PA Agent role thus emerged as a design response to themes related to staff engagement, competence development, and leadership support identified across domains (Fig 2).

Participants openly assessed which parts of the intervention elements could work and what needed to be adjusted. They pointed to the importance of maintaining flexibility and local adaptation. One member of the pedagogical staff expressed it in this way: “You have to be careful not to become too rigid (...) in relation to their children’s group.” (Staff, Kindergarten 4). A leader added: “We have 25 employees and have some different competencies (...) therefore, you have to make it [the intervention] your own.” (Leader, Kindergarten 3). These statements underscore that the intervention must relate to local differences in culture, competencies, and organization to function as sustainable structures for developing movement culture. Moreover, it underscored the theme of pedagogical judgment as a driving force.

### Design Experiment 2 (DE 2): Iterative Development of Practice and Competencies

DE 2 builds on the adjustments made in Lab 2 and included testing of playground time after lunch with appointed ‘PA boosters on the playground’ whose task was to initiate and support PA. Observations and informal field interviews with pedagogical staff indicated that this structure contributed to a high degree of PA participation among children, and the pedagogical staff were attentive to including children who were on the edge of the community: “They [pedagogical staff] both go in front and engage in dialogue with the children and actively involve children who are standing on the periphery.” (Staff, Kindergarten 3). This suggested that a structured approach may both strengthen pedagogical engagement, while also promoting inclusion and participation opportunities. These findings contributed to the continued development of structured playground roles as part of the final intervention design. This further illustrates how intervention elements were continuously refined through testing and reflection across domains (Fig 2).

### Lab 3: Development of competence development programs

Lab 3 was conducted to develop a multi-strand competence development program to strengthen pedagogical staff and leadership capacity for implementing and sustaining the intervention in everyday practice. The program ended up including: (1) an introductory meeting with the kindergarten leader and PA Agents to create shared understanding of the intervention’s goals and principles, (2) an introductory course for all staff with both practice-based and theoretical elements to ensure that the intervention becomes a shared task, (3) visits in the kindergartens to support implementation of the intervention elements, (4) a PA competence and facilitation course targeted at the PA Agents as resource persons in the kindergarten, and (5) a post-implementation network meeting for the PA Agents to create collegial learning in practice. Flexibility and context dependency were again prominent elements: “(...) It’s not about providing a one-size-fits-all solution like an introductory course for everyone; instead, you adapt it to get a clear picture of where the institution stands in relation to movement.” (Key stakeholder). Within this program, specific elements were designed to support PA Agents in their role as peer facilitators, particularly through targeted components such as the introductory meeting, the PA competence and facilitation course, and the network meetings, which focused on strengthening their ability to support and inspire colleagues in facilitating PA in everyday practice.

The multi-strand competence development program reflects findings from earlier phases, including observations and workshop discussions, where pedagogical staff and leaders emphasized the need for practical, context-adapted, and flexible training formats to support implementation in everyday practice, including structured training formats and opportunities for reflection and peer coaching. As illustrated in Fig 2, the competence development program consolidated insights generated throughout the iterative development process and linked these to the final intervention design.

### Reflections: Co-Design, Adoption, and Cultural Change

Although the Reflections domain culminated in the final group interviews, reflection was integrated throughout the development process and continuously informed the refinement of intervention elements across domains.

The intervention elements that were finally developed were:

- “Treasure chest” with PA words during active group time
- Kindergartens’ own play catalog
- key ring with pedagogical prompts
- Structured playground activities supported by ‘PA boosters’
- PA Agents
- A multi-strand competence development program

Fig 2 provides an overview of how these intervention elements were developed across domains and how they are expected to contribute to increased children’s PA through a set of interrelated mechanisms. The different elements of the intervention were designed to influence multiple, interrelated aspects of pedagogical practice and children’s participation. Specifically, the “treasure chest” was intended to function as inspiration for active group time, supporting children’s engagement in PA. The play catalog and key ring with pedagogical prompts were designed to support pedagogical staff’s practices on the playground. These elements were intended to strengthen the pedagogical staff’s ability to initiate, adapt, and reflect on PA, while also supporting child involvement and participation. The structured playground activities supported by ‘PA boosters’ and the PA Agent role were designed to strengthen everyday facilitation of PA by increasing adult engagement, peer support among staff, and attention to inclusion and participation opportunities for all children. Finally, the multi-strand competence development program was intended to build collective capacity among pedagogical staff and leaders to sustain and further develop PA, thereby supporting the development of a shared PA culture within the kindergarten.

Based on group interviews and observations, reflections on these intervention elements highlighted contextual factors and developmental processes that were pivotal for their adoption and sustainability. Co-design was consistently described as strengthening credibility and ownership by anchoring the intervention in pedagogical staff’s professional experiences rather than external prescriptions: “We actually came with our experiences as pedagogues. I think that makes a difference compared to if it was some smart person who said it.” (Staff, Kindergarten 4). The process also supported professional development by challenging existing practices: “It also made me go beyond my own limitations.” (Staff, Kindergarten 4).

Participants emphasized that intervention elements were continuously tested and adjusted within everyday practice, which increased their relevance and usability. Shared reflection and collegial knowledge exchange were highlighted as key mechanisms for developing practical PA solutions and strengthening collective competence: “We need to have some PA games to firefight with, and we find that out by talking with each other as colleagues across.” (Staff, Kindergarten 4).

A recurring theme was the gradual development of PA as an integrated pedagogical mindset rather than a set of isolated activities. PA was increasingly embedded within broader pedagogical goals such as inclusion and well-being: “The focus is on embedding PA into broader themes, including inclusion and well-being.” (Leader, Kindergarten 3). This shift required reflection on tacit professional practices, as staff became more aware of what they already did in everyday routines: “It puts me a bit to the test (…) to describe what you actually do, because tiger training is just something that is ingrained in me.” (Staff, Kindergarten 4). Participants emphasized that the intervention extended beyond the provision of isolated activities by foregrounding joy of PA as a core pedagogical value. Rather than focusing solely on specific games, the intervention sought to support broader changes in pedagogical practices and everyday opportunities for children’s participation in PA.

Further, leadership support emerged as crucial for sustainable adoption. Leaders highlighted that the intervention built on existing routines, making it easier to integrate into everyday leadership practices: “It makes it easier to lead on.” (Leader, Kindergarten 1). Moreover, leaders used PA-related tasks strategically to support staff development: “I get some pedagogues who grow from it. As a leader I can use it to boost some of my pedagogues.” (Leader, Kindergarten 1). Overall, sustainability was described as dependent on active leadership engagement, intervention flexibility, and local adaptation.

In sum, reflections emphasize how co-design, leadership engagement, and shared professional learning shaped a flexible yet structured intervention, supporting a shift from isolated activities to a pedagogical culture of PA. The relationships between intervention elements, expected mechanisms, and the intended outcome of increased children’s PA are summarized in Fig 2.

## Discussion

Across the development process, six overarching themes were identified: 1) PA as an overlooked pedagogical resource; 2) variation in children’s PA competences; 3) the role of leadership in cultural change; 4) pedagogical staff’s bodily engagement; 5) pedagogical judgment as a driving force; and 6) competence development as an adoption strategy.

Together, the analytical themes reflect central mechanisms and contextual conditions that informed the intervention design and are expected to support children’s participation in PA. They provide the foundation for the interventions theoretical underpinnings, intended mechanisms, and design decisions. As summarized in Fig 2, the analytical themes informed the intervention elements expected to influence children’s PA through changes in pedagogical practices, staff engagement, leadership support, and PA culture. The following section discusses each theme discussed in relation to intervention elements and implications for the upcoming feasibility phase.

### Theme 1: PA as an overlooked pedagogical resource

The initial context exploration revealed a fundamental tension. PA appears as an overlooked pedagogical tool while simultaneously holding significant potential for inclusion and community. This duality is well-known in literature, where barriers such as time, competencies, and lack of leadership support are often mentioned [9,10]. We took this duality into account by developing an intervention that opened possibilities for flexibility while keeping an eye on overarching structures through, for example, leadership support and the role as PA Agent. Several leaders and pedagogical staff in the study described how language learning activities and other political agendas often overshadow PA, even though children are “calling for something else.” This underscored the need to rethink PA as an integrated part of pedagogy. The testing of the “treasure chest” activity during group time illustrated how a simple structure had potential to function as a lever for both PA and language, which supported the points about practice-based and flexible approaches [12]. This connection between PA and learning goals (e.g., language) is central as it can legitimize the initiative in the kindergartens’ daily life, which is characterized by many different demands.

Taken together, these findings informed the development of intervention elements such as “treasure chest” during group time, which aim to integrate PA into everyday pedagogical routines. In the feasibility phase, it will be important to examine how such structured activities are implemented and whether they support sustained integration of PA in daily practice.

### Theme 2: Variation in children’s physical activity competences

The context exploration revealed substantial variation in children’s PA competencies across the participating kindergartens. Rather than representing an outcome of interest in itself, this variation was identified as an important contextual consideration that informed the development of flexible and adaptable PA opportunities. Observations during the design experiments showed how the same PA required different forms of support and adaptation across groups of children. The intervention’s emphasis on flexible materials and adaptable activities directly responded to this finding. This aligns with literature emphasizing the importance of designing interventions that accommodate heterogeneous groups [32].. These findings informed the development of flexible and adaptable intervention elements, such as the play catalog and differentiated activities, designed to accommodate variation in children’s PA competencies and thus support participation in PA.

From an equity perspective, this variation highlights the importance of how pedagogical practices can be organized to accommodate diverse competencies. In this study, such variation is not assessed at the level of individual children but is reflected in how pedagogical staff describe and respond to differences in children’s engagement. The findings therefore point to the role of flexible structures and professional judgment in shaping conditions that may support broader access to PA.

In the feasibility phase, it will be relevant to examine how these elements function in practice, including whether they effectively support inclusive participation and provide equitable access to PA opportunities.

### Theme 3: The role of leadership in cultural change

The results from the development phase underscore the critical importance of leadership in driving cultural change around PA in kindergartens. In line with this, a significant strength of the process was that leaders participated in the design workshops on equal footing with the pedagogical staff. This created shared understanding and organizational anchoring, which is crucial for implementation and sustainability [10,20]. The development of the “PA Agents” highlights a well-known dilemma: How is the need for continuity balanced with the desire for broad ownership? Our data show that when responsibility is concentrated among few people, the collective ownership is weakened. However, without clear roles and responsibilities, the integration of PA in kindergartens could be underprioritized [12]. Observations in DE 2 showed, for example, that agreed activities often failed to materialize when those responsible were not present. These findings underscore that cultural change cannot rest on individuals but requires collective processes. In response to this, the intervention was developed using a multi-strand competence development program where multiple PA Agents in the same kindergarten should function as resource persons but maintaining a focus on collegial consultation and shared responsibility. All pedagogical staff should be involved in strengthening collective ownership and ensure that PA becomes an integrated part of daily life. This is consistent with other studies showing that change is only anchored through either strong leadership/external support or collective capacity building, which is crucial for implementation quality [6,7].

This theme directly informed the inclusion of leadership involvement and the PA Agent role as key elements of the intervention. In the feasibility phase, it will be important to examine how leadership engagement is enacted in practice, including how roles, responsibilities and support structures contribute to implementation and sustainability.

### Theme 4: Pedagogical staff’s bodily engagement

Observations showed that pedagogical staff’s bodily engagement was central to children’s participation in PA games. When pedagogical staff participated actively and showed joy of PA, there was potential to increase children’s motivation and inclusion. This is consistent with two reviews showing that PA interventions are often challenged by lack of support from professionals who lack PA-related competencies [6,33]. These reviews support that structured techniques with support and coaching of professionals combined with variation and playful elements increase the effect on children’s PA participation. The results are consistent with previous research pointing to the necessity of supporting pedagogical staff as competent actors when implementing PA interventions [11,17,18,26]. In line with this, the study has focused on creating a practice-based and adaptive approach where early involvement of key stakeholders and co-design with leaders and pedagogical staff have been central elements. In this way, the project followed the MRC guidance for complex intervention developing and evaluating, emphasizing the adaption of interventions to local contexts and foster shared responsibility to strengthen implementation [20].

These findings informed intervention elements that emphasize adult participation and modeling in PA activities. In the feasibility phase, it will be important to assess how such engagement is enacted in practice and how it influences children’s participation in PA.

### Theme 5: Pedagogical judgment as a driving force

A central finding was how pedagogical staff’s professional judgment became a driving force in the intervention’s implementation. During the design experiments, pedagogical staff continuously made situational assessments about when, how, and with whom to integrate PA activities. These judgments were based on their knowledge of individual children, group dynamics, and daily rhythms in the kindergarten. Pedagogical staff exercised pedagogical judgment to adapt activities to emerging situations, demonstrating that successful PA integration requires professional discretion and contextual sensitivity. The reflection domain in our results reinforces this, as participants emphasized that co-designing materials in practice with colleagues strengthened credibility, ownership, and professional growth [34]. Testing and adjusting the intervention elements in daily routines made them authentic tools rather than theoretical constructs, supporting sustainability and adoption.

Taken together, these findings highlight that the intervention combines structured elements with flexibility, supporting pedagogical staff in making context-sensitive decisions within everyday situations. In this sense, pedagogical judgment can be understood as a key mechanism through which pedagogical practices and PA opportunities are shaped to support children’s participation in PA.

This theme informed the development of flexible tools such as the key ring and play catalog, which support situational adaptation. In the feasibility testing, it will be important to examine how such tools support or potentially constrain, professional judgment across different contexts and how they are used to balance flexibility with consistency in practice.

### Theme 6: Competence development as an adoption strategy

Competence development became a central track in the intervention, where collaborative reflection and practice-based learning should support implementation. It was a recurring theme during the design process that participants both evaluated the intervention elements and critically assessed their implementation in everyday practice. Results from a recent study show that a flexible and practice-based approach with active involvement of pedagogical staff can lead to some measurable improvements in children’s PA levels [32]. Further, competence development, resources, and coaching are highlighted in literature as central to making PA an integrated part of the institution’s culture [35]. This approach ensured that the intervention was not imposed from outside but grew organically from the kindergartens’ own contexts and needs.

This theme informed the multi-strand competence development program, including training, coaching, and reflection activities. The feasibility phase will be essential for examining how these elements support implementation, competence development, and intervention quality.

### Implication for practice

Across themes, the findings highlight that the intervention is not defined by isolated activities, but by the interplay between structured elements and pedagogical processes. This reflects a shift from activity-based approaches towards supporting a broader PA culture in kindergarten practice. This also has implications for how the intervention can be transferred to other settings. An important consideration for transferability is the distinction between core intervention elements and elements intended for local adaptions. In the present study, the core elements of the intervention include: “treasure chest” with PA words during active group time; kindergartens’ own play catalog; key ring with pedagogical prompts; structured playground activities supported by ‘PA boosters’; designation of PA Agents, and; a multi-strand competence development program.

In contrast, the specific content of activities, the organization and enactment of roles such as PA Agents, and the ways in which elements and materials (e.g., the play catalog and prompts) are applied are intended to be flexible and adapted to local contexts, children’s needs, and existing pedagogical practices. In addition, key pedagogical processes, such as staff’s active bodily engagement and use of professional judgment, are expected to vary across contexts and are supported rather than prescribed by the intervention elements.

Taken together, this distinction highlights a deliberate balance between structure and flexibility in the intervention design. This balance is central to supporting both implementation and local ownership and reflects the context-sensitive nature of DBR.

Overall, these mechanisms illustrate how participatory, contextually grounded and professionally anchored design processes can strengthen the kindergarten as an early‑childhood public health setting by building institutional capacity for sustainable, PA‑rich environments.

### Strengths and Limitations

One of the most striking strengths of Moving Kindergartens is the close connection between research and practice. The intervention was developed in iterative co-design processes where pedagogical staff, leaders, and researchers together formulated, tested, and adjusted the content. This created a high degree of practice relevance, and strengthened the intervention’s legitimacy and ownership in practice, which is crucial for sustainability [36]. The study also benefited from the DBR approach, which enabled continuous adaptation of intervention elements in response to stakeholder feedback and contextual conditions. Through iterative cycles of development and testing, the intervention was grounded in everyday kindergarten practice while remaining sufficiently flexible to accommodate local variation across settings.

At the same time, several limitations should be acknowledged. First, the co-design approach may have introduced a degree of bias, as participants who engaged in the development process may have had a particular interest in PA or been more motivated to contribute to change processes.

Second, the active role of researchers in facilitating workshops, observations, and iterative development processes may have influenced both the co-design process and the interpretation of the data. While this is inherent to DBR it requires careful consideration of researcher reflexivity.

Third, the study is context-specific and was conducted in a limited number of Danish kindergartens. Although the intervention was developed to be adaptable across settings, transferability should be considered in relation to local organizational conditions, cultures, and participant characteristics. More broadly, DBR seeks to generate usable knowledge that can inform practice across settings while remaining responsive to local context. Balancing contextual relevance and broader applicability therefore remains an ongoing challenge.

Fourth, children were not directly involved as co-design participants. Although children’s perspectives informed the process indirectly through pedagogical staff’s observations of children’s participation and responses to PA, direct involvement of children may have generated additional insights. The study deliberately focused on pedagogical staff and leaders as key actors responsible for organizing, facilitating, and sustaining PA practices in kindergarten settings.

Finally, the present study focuses exclusively on the intervention development phase and does not evaluate intervention effectiveness. The extent to which the intervention contributes to increased PA and related outcomes remains to be examined through future feasibility and evaluation studies.

## Conclusion

This article examined the co-design process forming the foundation for a kindergarten-based PA intervention using a DBR approach. Development unfolded through iterative cycles across four domains: Context, Lab, Design Experiments, and Reflections – with participation from key stakeholders, pedagogical staff, leaders, and researchers.

The analysis shows that developing a PA intervention was not only about creating activities but about establishing shared understanding and legitimacy. First, the process shifted perceptions of PA from an isolated task to an integrated pedagogical resource within everyday practice. Second, flexible intervention materials supported the adaptation of PA opportunities to diverse groups of children. Third, leadership support was essential for prioritizing and sustaining PA-related practices. Fourth, pedagogical staff’s bodily engagement played a key role in integrating PA into daily routines. Fifth, professional judgment enabled local adaptation while maintaining the intervention’s pedagogical intentions. Finally, systematic competence development supported the integration of PA as a shared pedagogical principle.

These findings demonstrate how participatory, settings-based approaches can support the development of PA-rich environments within kindergarten practice. From a practical perspective, the findings suggest that kindergarten-based PA initiatives may benefit from combining leadership involvement, competence development, locally adaptable intervention elements, and active engagement of pedagogical staff throughout the development process. Together, these insights provide a foundation for the upcoming feasibility study, which will test the implementation of the finalized intervention and evaluation procedures in kindergartens. The next phase bridges development and evaluation and contributes to evidence on designing and implementing complex PA interventions in kindergarten settings.

## Supporting information

S1 File COREQ Checklist. (PDF)
